# Silicon enhancement of nitrogen use efficiency in plants: insights from soil nitrogen transformation, plant nitrogen uptake, assimilation, and remobilization

**DOI:** 10.3389/fpls.2026.1893351

**Published:** 2026-08-13

**Authors:** Zina Li, Tianyu Wang, Jianli Jimu, Yuan Liu, Wenbing Li, Huachun Sheng

**Affiliations:** 1 College of Pharmacy and Food, Southwest Minzu University, Chengdu, Sichuan, China; 2 Tibetan Plateau Ethnic Medicinal Resources Protection and Utilization Key Laboratory of National Ethnic Affairs Commission of the People’s Republic of China, Southwest Minzu University, Chengdu, Sichuan, China; 3 Sichuan Provincial Qiang-Yi Medicinal Resources Protection and Utilization Technology and Engineering Laboratory, Southwest Minzu University, Chengdu, Sichuan, China

**Keywords:** nitrogen remobilization, nitrogen transformation, nitrogen use efficiency, silicon, soil microbial community

## Abstract

Severe nitrogen (N) deficiency inhibits crop growth and yield, while low use efficiency of N fertilizer causes resource waste and environmental harm. Enhancing crop N use efficiency (NUE) is thus a critical scientific challenge. Ecofriendly and cost-effective silicon (Si) fertilizer can enhance NUE across the entire soil-plant continuum. Si-mediated improvements in soil physicochemical properties and microbial community structure underlie enhanced mineralization and suppressed denitrification. Si–cell wall interactions support symbiont recognition, nodule initiation, and formation of functional nodules for sustained N_2_ fixation. Si also upregulates genes encoding N transporters and assimilatory enzymes to improve N uptake, assimilation, and remobilization. This mini-review synthesizes Si’s roles in regulating soil N transformation and plant N uptake, assimilation, and remobilization. It aims to support precision Si fertilization strategies that reduce synthetic N input without compromising yield.

## 1 Introduction

Nitrogen (N) is one of the essential macronutrients for plant growth and development, with its content in plants typically ranking fourth, following carbon, hydrogen, and oxygen (Brown et al., 2022). As a core component of proteins, nucleic acids (DNA and RNA), chlorophyll, enzymes, hormones, and various secondary metabolites, N directly underpins key physiological processes including photosynthesis, energy metabolism, cell division, and signal transduction (Nunes-Nesi et al., 2010; Liu et al., 2025). Plants primarily acquire inorganic N via their root systems, with ammonium (NH_4_^+^) and nitrate (NO_3_^-^) as the main forms. A subset of symbiotic N-fixing plants (e.g., legumes) can further convert atmospheric dinitrogen (N_2_) into bioavailable ammonia through symbiosis with rhizobia (Xu and Wang, 2023). Soil N exists in diverse forms, and its bioavailability is highly dependent on microbially mediated biogeochemical processes, including mineralization, nitrification, denitrification, and N_2_ fixation (Kuypers et al., 2018). N bioavailability is also regulated by factors such as soil pH, moisture, temperature, organic matter content, and agronomic management practices (Ros et al., 2011; Kader et al., 2013). N deficiency often manifests as stunted growth, plant senescence, reduced photosynthesis and leaf area, and significant yield decline (Mu and Chen, 2021; Fan et al., 2023). Conversely, excessive N application may induce deteriorated fruit quality (Xiong et al., 2026) and impaired stress tolerance (Ye et al., 2022).

N is predominantly supplied via chemical fertilizers, and global N fertilizer consumption surged from 10 million tons in 1961 to 110 million tons in 2020 (Rajonee et al., 2026). More alarmingly, despite the consistent increase in N fertilizer application over the past decades, the yields of staple grain crops on 24% to 39% of the world’s arable land have not shown a corresponding upward trend (Xia and Yan, 2023). This implies a low NUE (approximately 40%), with significant quantities of N being lost from the soil, which leads to economic losses and severe environmental concerns including eutrophication, acid rain, and ozone layer depletion (Robertson and Vitousek, 2009; Xia and Yan, 2023; Rajonee et al., 2026). Thus, the scientific regulation of N supply and enhancement of NUE have emerged as critical components for developing the sustainable modern agriculture and establishing the green production systems.

Silicon (Si) is a plant-beneficial quasi-essential element with well-documented critical physiological functions. While it not yet classified as an essential nutrient for higher plants, extensive empirical research demonstrates that Si exerts pronounced effects across diverse monocotyledonous and dicotyledonous crops by significantly promoting growth and development, enhancing tolerance to biotic and abiotic stresses, and improving yield components and quality traits (Coskun et al., 2019; Mandlik et al., 2020; De Tombeur et al., 2023). Si can regulate the homeostasis of other mineral elements in plants (Sheng et al., 2024; Costa et al., 2024a; Pavlovic et al., 2021; Hailai et al., 2024), with particularly prominent regulatory roles in N metabolism (Sheng et al., 2018). Field trial results indicate that supplementary Si fertilization, when applied alongside conventional nutrient management, enables a reduction in N fertilizer input while maintaining or increasing crop yield (Galindo et al., 2021; Barão, 2023; Wu et al., 2025). This translates to a marked improvement in NUE, thus the integration of Si into integrated nutrient management frameworks carries substantial scientific significance. However, to improve NUE, most agronomists have focused on advanced field N management practices (Gu et al., 2023), while research on strategies substituting Si fertilizer for N fertilizer remains insufficient. In this mini-review, we aim to systematically synthesize and critically evaluate the multifaceted mechanisms through which Si nutrition enhances NUE across the entire soil-plant continuum, thereby offering robust theoretical support for developing the green cultivation strategies that reduce N input while improving NUE via Si nutrition management.

## 2 Si enhances plant-available N in soil through promoting mineralization and inhibiting denitrification

The total N content in most soils ranges from 200 to 4000 ppm, of which approximately 90% exists in organic forms, while merely 1–4% is present as inorganic nitrate and ammonium forms (Lindsay, 1979; Olk, 2015). Organic N can be transformed into inorganic N through microbial-mediated mineralization, a biogeochemical process highly sensitive to soil pH. Globally, soil microbial biomass is the dominant driver of N mineralization, exerting a greater effect magnitude than climate and soil physicochemical properties (Li et al., 2019). Moreover, across two agricultural field sites, soil microbial biomass increased significantly with rising pH across the range of 3 to 7 (Kemmitt et al., 2006). Si fertilizers commonly comprise soluble metasilicates (such as sodium metasilicate) and are classified as Lewis bases; theoretically, they can elevate soil pH. From the plant perspective, Si can also downregulate the expression levels of proton pump-encoding genes and organic acid secretion-related genes in rice plants, thereby elevating rhizosphere pH (Pang et al., 2023). These Si-induced elevations in soil pH may consequently promote soil microbial biomass accumulation, thereby enhancing N mineralization. As expected, it has demonstrated that Si fertilizer application significantly increases soil microbial populations and enhances soil N availability in maize rhizosphere (Rangaraj et al., 2014).

The initial step of N mineralization is ammonification, a microbial enzyme-catalyzed process that produces ammonium and functions as the rate-limiting step of N mineralization. Ammonium is sequentially converted to nitrite and then to nitrate via nitrification, where each step of this aerobic oxidation of ammonium is mediated by distinct groups of specialized bacteria (*Nitrosomonas* and *Nitrobacter*) (Benbi and Richter, 2002). Specifically, in paddy soil, Si can significantly increase the abundance of ammonia-oxidizing bacteria (AOB), key functional taxa mediating the nitrification of ammonium (Liang et al., 2021). Conversely, nitrate is sequentially reduced to nitrite, nitric oxide, nitrous oxide (N_2_O), and N_2_ via denitrification, a process of anaerobic respiration coupled to the oxidation of organic carbon as well as reduced iron/sulfur species (Thamdrup, 2012). Currently, the majority of studies indicate that Si fertilizer application inhibits soil denitrification. Si-based fertilizer application to soil has been shown to suppress N_2_O emissions across diverse agricultural systems (Wang et al., 2023; Tang et al., 2026; Xu et al., 2026; Hoffmann et al., 2025; Yang et al., 2025). For instance, in acidic soils under tea cultivation, Si fertilizer amendment concurrently attenuates both N_2_O and CO_2_ emissions, indicating coordinated suppression of N reduction and microbially driven organic carbon oxidation during denitrification (Wang et al., 2023). A plausible mechanism is that Si fertilizer input enhances soil porosity and aeration, thereby increasing soil oxygen concentration, which likely inhibits microbial denitrification (Madegwa et al., 2025).

Very recently, Qin et al. (2026) reported that Si significantly reduces denitrification rates and suppresses both N_2_O production and its subsequent transformation by restructuring soil microbial community composition, specifically through the enrichment of beneficial bacterial taxa within the genus *Bacillus*. The genus *Bacillus*, a key participant in soil elemental redox cycling, exhibits susceptibility to community structural restructuring in response to soil pH fluctuations (Wang et al., 2025, Wang et al., 2026), suggesting that Si facilitates the enrichment of *Bacillus* maybe through modulating soil pH and then reduces denitrification rates. Meanwhile, Si-induced shifts in microbial community structure are further corroborated by downregulating functional denitrification genes, namely *nirS*, *nirK*, and *nosZ* (Song et al., 2017).

Notably, the application of Si fertilizer may exert adverse effects under specific circumstances. For instance, under severe waterlogging (extremely anaerobic) conditions, while Si fertilization can enhance the waterlogging tolerance of barley, it facilitates a more complete denitrification process, leading to the increased N_2_ emission (Włodarczyk et al., 2019). This implies that following the recession of flooding, barley plants receiving Si fertilization may suffer from N deficiency. In contrast to the inhibitory effect of Si in acidic tea plantation soils (Wang et al., 2023), its promotion of denitrification in waterlogged soils suggests that the role of Si is environment-dependent.

**Figure 1:**
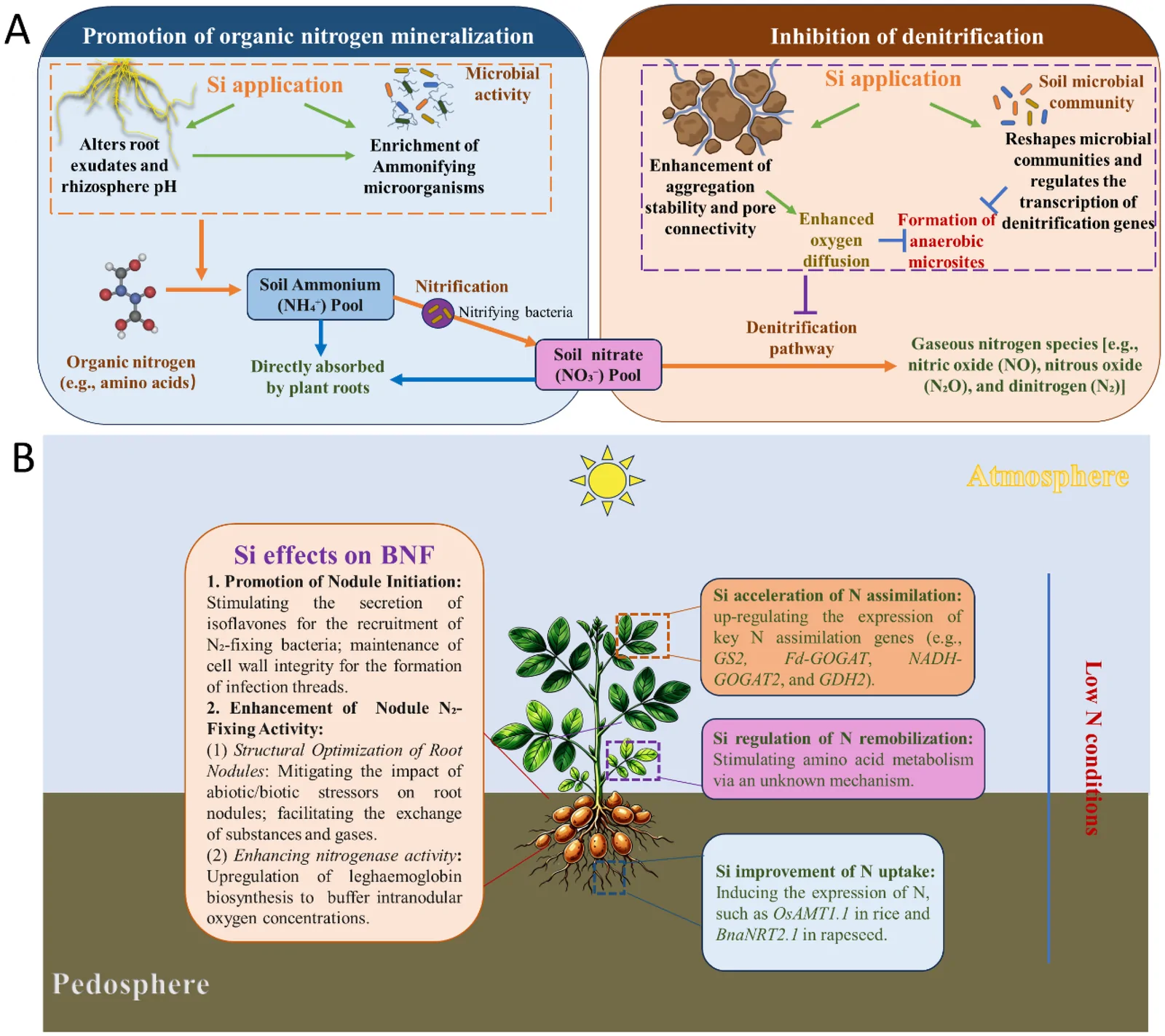
The mechanisms by which Si promotes NUE. **(A)** Si-based fertilizers enhance soil N availability. The application of Si fertilizer enhances mineralization processes. As a Lewis base, Si fertilizer can elevate soil pH; furthermore, Si inhibits the secretion of organic acids by plants and the expression of proton pumps in plants, thereby increasing rhizosphere pH. The rise in soil pH promotes an increase in the abundance of soil microorganisms (including ammonifying bacteria), which facilitates the conversion of organic N into plant-available ammonium. Si inhibits denitrification. The application of Si fertilizer improves soil porosity and aeration, thereby increasing soil oxygen concentration. Si can also reshape the soil microbial community and downregulate functional denitrification genes, namely *nirS*, *nirK*, and *nosZ*. These effects ultimately suppress the formation of anaerobic microsites, thereby inhibiting the conversion of nitrate N to gaseous N and reducing associated N loss. **(B)** Si promotes N_2_ fixation, as well as plant N uptake, assimilation, and remobilization. Si enhances root nodule formation and N_2_-fixing activity mainly in leguminous plants. Under low N conditions, Si-mediated enhancement of N uptake, assimilation, and remobilization suggests that precision application of Si fertilizers can improve crop yield while minimizing N input requirements and environmental trade-offs.

Overall, Si enhances plant-available N in soil by promoting organic N mineralization and suppressing denitrification (**Figure 1A**). Specifically, Si-mediated alterations in root exudation and rhizosphere pH stimulates microbial activity (particularly that of ammonifying microorganisms), thereby accelerating the conversion of complex organic N compounds (including amino acids, proteins, and chitin) into NH_4_^+^, the primary inorganic form directly assimilated by plant roots or subsequently oxidized to NO_3_^-^ via nitrification. Concurrently, Si reduces N loss via denitrification by improving soil physical structure, increasing soil pH, and then reshaping the soil microbial community. These changes enhance oxygen diffusion, limit the formation and persistence of anaerobic microsites, and modulate the transcription of key denitrification genes (e.g., *nirK*, *nirS*, *nosZ*) in select bacterial populations. Together, these complementary mechanisms that increase N supply from organic reservoirs while minimize gaseous N losses, enhance soil NUE and support improved crop N nutrition, particularly under abiotic stress or suboptimal management conditions.

## 3 Si fertilizer application promotes biological N_2_ fixation

Beyond anthropogenic application of chemical N fertilizers, the conversion of atmospheric N_2_ to plant-available NH_4_^+^ via symbiotic bacteria represents another core N input source in agricultural ecosystems (Xu et al., 2012). The total annual N input through biological N fixation (BNF) across global agricultural systems is approximately 50–70 teragrams (Tg) (Herridge et al., 2008). At present, research on Si fertilization-enhanced BNF has been primarily concentrated on leguminous plants. Extensive empirical evidence indicates that Si supplementation enhances symbiotic N fixation in legumes through the coordinated regulation of three critical processes: nodule initiation, structural development, and physiological function (Putra et al., 2020).

*Si Promotion of Nodule Initiation*. The initial phase of root nodule formation hinges on precise chemical signaling crosstalk between leguminous plants and rhizobia. Si acts as a “signal enhancer and stabilizer” in this symbiotic interaction. Si supplementation improves root morphological traits and stimulates the secretion of signaling molecules (e.g., isoflavonoids) from root tissues (Tripathi et al., 2021). These compounds effectively chemoattract free-living rhizobia in the rhizosphere, and then the biosynthesis of nodulation factors (Nod factors) was activated by rhizobia (Nelwamondo and Dakora, 1999; Tripathi et al., 2021). Moreover, the extension of infection threads relies on the directional expansion of host cell walls, and thus maintaining the structural integrity of the cell wall is critical during infection thread development (Zhao et al., 2025). Our previous studies have demonstrated that Si nutrition enhances cell wall integrity via covalent cross-linking with cell wall components (Sheng and Chen, 2020; Sheng et al., 2023). Therefore, Si fortifies the cell walls of root hair cells, potentially providing a more robust conduit for the formation of infection threads during rhizobial invasion. This enhancement elevates the efficiency of early nodulation events, including root hair curling and cortical cell division, ultimately leading to a significant increase in nodule number per plant following Si application.

*Si Enhancement of Nodule N_2_-Fixing Activity.* Upon uptake, Si is specifically deposited as amorphous silica at key sites within root nodules (Putra et al., 2020). This silicification confers dual structural optimization effects on root nodules. Firstly, Si deposition enhances cell wall mechanical strength and rigidity (Sheng and Chen, 2020), which significantly improves nodule tolerance to soil mechanical stress, pest and pathogen infestation, and other biotic and abiotic stresses, establishing a stable physical microenvironment conducive to N_2_ fixation (Putra et al., 2020). Secondly, rather than forming an impermeable barrier, Si deposition fine-tunes the microporous architecture of cell walls (Nelwamondo et al., 2001; Garg and Singh, 2018), thereby increasing nodule permeability. This modulation critically results in the rapid transport of solute and gasses between nodules, root tissues and the soil (Putra et al., 2020).

The N_2_-fixing activity of root nodules, centered on nitrogenase activity as the primary functional index, directly governs the efficiency of BNF. It has been reported that Si supply promotes nitrogenase activity (Putra et al., 2021). Given the strict oxygen sensitivity of nitrogenase and the obligate requirement of bacteroids for aerobic respiration, Si application enhances nodule N_2_-fixing activity (nitrogenase activity) in two pigeonpea genotypes through the upregulation of leghaemoglobin biosynthesis (Garg and Singh, 2018). Because symbiotic leghaemoglobin serves a regulator of intranodular oxygen concentrations, thereby (i) maintaining microaerobic conditions to stabilize nitrogenase activity against oxidative inactivation; and (ii) facilitating controlled oxygen diffusion to support bacteroid respiratory metabolism (Ott et al., 2005).

Recent studies conducted on *Trifolium incarnatum* have demonstrated that Si supplementation not only increases nodule number, elevates the relative abundance of nitrogenase, and enhances N_2_ fixation activity, but also induces significant alterations in the root proteome of the host plant and the proteome of bacteroids (Coquerel et al., 2024). In the same study, analysis of isolated nodules revealed the accumulation of certain elements critical to nitrogenase function, such as sulfur (Coquerel et al., 2024).

In summary, Si fertilizer application does not directly supply a N source; instead, it functions as a “systemic facilitator” and “structural stabilizer” that comprehensively supports and enhances the BNF capacity of leguminous plants (**Figure 1B**). Specifically, it facilitates symbiotic partner recognition and nodule formation, constructs robust and functionally specialized nodule structures, and ensures the sustained, efficient operation of N_2_-fixing biochemical reactions.

## 4 Plant Si accumulation enhances N uptake, assimilation and remobilization

In aerobic soils, NO_3_^-^ is the predominant inorganic N species; in contrast, NH_4_^+^ dominates under flooded (e.g., paddy) or strongly acidic soil conditions (Xu et al., 2012). Upon uptake, NO_3_^-^ is reduced to NO_2_^-^ by nitrate reductase (NR) in the cytosol, followed by its translocation to chloroplasts where nitrite reductase (NiR) catalyzes the conversion of NO_2_^-^ to NH_4_^+^ (Zhang et al., 2025). Subsequently, NH_4_^+^ is assimilated into glutamate (Glu) predominantly via the glutamine synthetase/glutamate synthase (GS/GOGAT) pathway, with the glutamate dehydrogenase (GDH) pathway contributing under specific physiological conditions such as high NH_4_^+^ availability or stress (Vega-Mas et al., 2019).

Si fertilizer helps maintain plant N balance under varying N supply. In the Si accumulator of rice, Si up-regulates key N assimilation genes (e.g., *OsGS2, OsFd-GOGAT*) under low-N conditions, enhancing N assimilation; however, its effect on N uptake remains inconclusive due to opposing regulation of *OsNRT1 (OsNPF8.9)* and *OsAMT1.1* (Wu et al., 2017). Subsequent studies showed Si promotes NH_4_^+^ uptake in rice by modestly increasing OsAMT1.1 protein abundance (~1.14-fold) and boosting OsGDH2 levels (Sheng et al., 2018). In contrast, in non-accumulator rapeseed, Si enhances N acquisition under low-N conditions primarily by up-regulating the high-affinity nitrate transporter *BnaNRT2.1*, not *BnaAMT1.1* (Haddad et al., 2018). Under high-N conditions, Si exerts divergent effects on N uptake and assimilation across plant species: it suppresses both processes in rice by down-regulating key N-related genes (Wu et al., 2017), yet enhances N assimilation and chlorophyll synthesis in cucumber via activation of N-metabolizing enzymes and up-regulation of related genes (Gou et al., 2020).

Based on the aforementioned studies, it is evident that Si modulates the expression of genes associated with N uptake and assimilation in a plant species-specific manner, irrespective of N supply levels. While Si exhibits inconsistent effects under high-N conditions, N deficiency remains the prevalent state in crop production systems. Notably, nearly all existing studies have demonstrated that Si consistently enhances N uptake and assimilation across diverse plant species under low-N conditions (Costa et al., 2024b; Parecido et al., 2022; Mabagala et al., 2020; Deus et al., 2020), suggesting that reducing N input while increasing NUE via appropriate Si fertilizer application represents a viable strategy (**Figure 1B**).

During the vegetative growth stage, leaves function as N sinks; upon entering the senescence phase, N is predominantly remobilized in the form of amino acids to developing grains (Xu et al., 2012). It has been reported that Si nutrition enhances grain yield in rice even under non-stress conditions, concomitant with improved NUE (Detmann et al., 2012). Furthermore, Si supplementation induces significant alterations in primary metabolism, particularly promoting the remobilization of amino acids (Detmann et al., 2012). Furthermore, Si supplementation can delay leaf senescence in plants grown under N-deficient conditions and/or open-field cultivation, while also enhancing the uptake of N from fertilizers (Haddad et al., 2018; Laîné et al., 2019). Extending the lifespan of the leaves could allow for better synchronization between the remobilization of foliar N and the period of N accumulation in the seeds.

Taken together, Si improves NUE by promoting N uptake and assimilation via transcriptional regulation, as well as stimulating N remobilization. Unfortunately, the molecular and physiological mechanisms governing this process remain incompletely characterized. Recent studies show that Si regulation of pectin methyl-esterification and cellulose microfibril organization modulates FERONIA homologs OsFLR1/2-mediated cell wall integrity signaling for rice adaptation to salt stress (Lei et al., 2025). Additionally, the RALF1-FERONIA complex interacts with and activates TOR signaling in response to low N supply in Arabidopsis (Song et al., 2022). It is hypothesized that Si-induced transcriptional regulation under low-N conditions may also be associated with FER-mediated cell wall integrity signaling, though this assumption warrants further validation.

## 5 Conclusions and future perspectives

**Figure 2:**
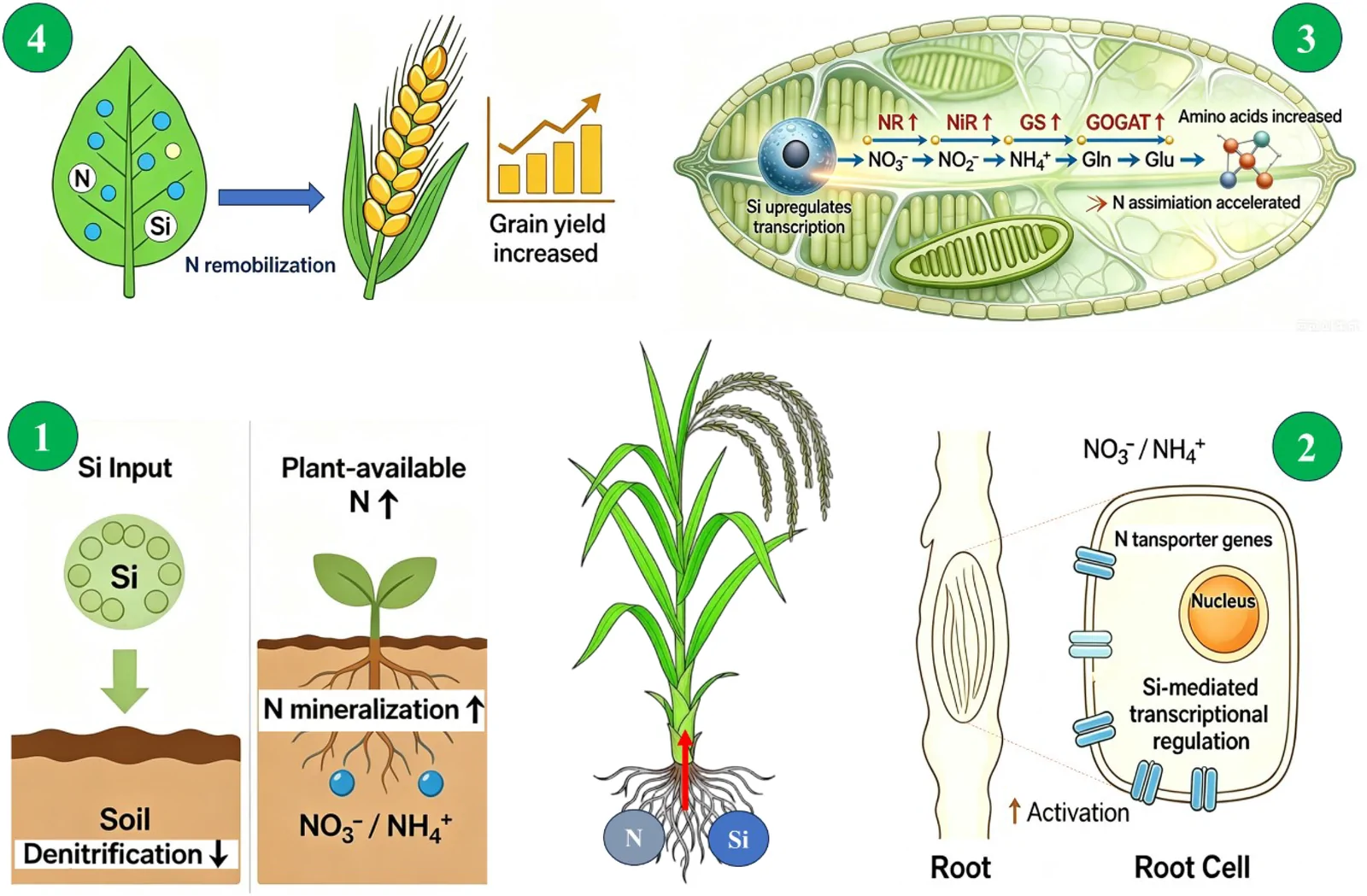
Si regulates NUE along the entire soil-root-plant-grain pathway. (1) In soil, Si fertilizer enhances the plant-available N by facilitating N mineralization and inhibiting denitrification. (2) Si modulates the expression of N transporters in plant roots, thereby enhancing N uptake by plants. (3) Si enhances N assimilation in plant leaves through transcriptional regulation of the GS-GOGAT cycle. (4) Si promotes N remobilization via mechanisms that remain to be elucidated.

This review summarizes the potential mechanisms by which Si nutrition enhances NUE throughout the soil-root-plant-grain regulatory pathway (**Figure 2**), including: (1) elevating the concentration of plant-available N via promoting organic N mineralization and BNF; (2) mitigating soil N loss through the inhibition of denitrification; and (3) enhancing plant N uptake, assimilation, and remobilization. Notably, the enhancement of mineralization and suppression of denitrification are primarily attributed to Si-mediated improvements in soil physicochemical properties and reshaping of the soil microbial community. The promotion of BNF is likely linked to Si-cell wall interactions: Si deposition on the cell wall or its cross-linking with cell wall components not only facilitates symbiont recognition and nodule initiation but also contributes to the formation of structurally robust and functionally specialized nodules, thereby ensuring the sustained, efficient progression of N_2_-fixing biochemical reactions. Moreover, Si enhances plant N uptake and assimilation through transcriptional regulation of genes encoding N transporters and key assimilatory enzymes, and promotes N remobilization via mechanisms that remain to be elucidated.

Research on plant nutrient signal transduction has emerged as a cutting-edge focus in the fields of plant physiology and crop nutrition. Notably, the perception, initiation, and downstream transduction pathways of N nutrient signals have been relatively systematically elucidated. In contrast, the molecular underpinnings of Si nutrient signals remain largely uncharacterized, with related research still in its nascent stage (Lei et al., 2025). Thus, future studies urgently need to focus on the cross-regulatory mechanisms between N and Si nutrient signaling pathways. Additionally, existing research lacks a systematic elaboration of the physiological and molecular mechanisms by which Si regulates N remobilization, as well as an exploration of the slow-release and synergistic effects of Si nanoparticles or Si-based composite materials on improving NUE. Breakthroughs in these research directions will facilitate a deeper understanding of the core physiological and molecular mechanisms through which Si reduces external N fertilizer input while enhancing crop NUE.
